# Deep Learning Techniques in Intelligent Fault Diagnosis and Prognosis for Industrial Systems: A Review

**DOI:** 10.3390/s23031305

**Published:** 2023-01-23

**Authors:** Shaohua Qiu, Xiaopeng Cui, Zuowei Ping, Nanliang Shan, Zhong Li, Xianqiang Bao, Xinghua Xu

**Affiliations:** National Key Laboratory of Science and Technology on Vessel Integrated Power System, Naval University of Engineering, Wuhan 430033, China

**Keywords:** fault diagnosis, fault prognosis, machine learning, deep learning, industrial systems

## Abstract

Fault diagnosis and prognosis (FDP) tries to recognize and locate the faults from the captured sensory data, and also predict their failures in advance, which can greatly help to take appropriate actions for maintenance and avoid serious consequences in industrial systems. In recent years, deep learning methods are being widely introduced into FDP due to the powerful feature representation ability, and its rapid development is bringing new opportunities to the promotion of FDP. In order to facilitate the related research, we give a summary of recent advances in deep learning techniques for industrial FDP in this paper. Related concepts and formulations of FDP are firstly given. Seven commonly used deep learning architectures, especially the emerging generative adversarial network, transformer, and graph neural network, are reviewed. Finally, we give insights into the challenges in current applications of deep learning-based methods from four different aspects of imbalanced data, compound fault types, multimodal data fusion, and edge device implementation, and provide possible solutions, respectively. This paper tries to give a comprehensive guideline for further research into the problem of intelligent industrial FDP for the community.

## 1. Introduction

### 1.1. Background

Industrial systems are typical complex systems with various subsystems and device types of mechanical system, power system, information system, electronic system, or their combinations. They are playing an increasingly important role in the economy, such as manufacturing industry, energy industry and chemical industry, which are now developed with more functions, more sophisticated structures, and larger scales [1]. Reliability issues have gradually become the key of whether many modern industrial systems can be truly practical. Once a failure occurs, it may affect the safe and stable operation of the entire system, i.e., reducing the efficiency of the system, and causing system breakdown or damage in severe cases [2]. It may also endanger personnel safety, and cause other catastrophic consequences. Therefore, the early identification of faults in advance can greatly help to take appropriate actions of maintenance to avoid the undesired consequences.

Driven by demand, prognostics and health management (PHM) [3] technology, firstly originated from engine health monitoring systems [4], has gained increasingly more attention. PHM is an expansion of the traditional reliability or predictive maintenance concept oriented for complex industrial systems. It realizes the development from the initial condition monitoring and fault diagnosis that aims to estimate health status, to health management that aims at formulating the countermeasures based on the results of monitoring, diagnosis, and prognosis.

In practical scenes, it is often difficult or even impossible to establish mathematical models of complex components or systems [5], in order to trace and analyze faults. Therefore, a large amount of historical data that were collected in the process of system operation and maintenance have become the major method by which to evaluate the system’s health status. As the core part of PHM technology, the fault diagnosis and prognosis (FDP) technique based on data-driven machine learning (ML) methods recognizes or learns the health features of the system from historical data, and tries to discover and mine the information hidden in the data, so that it can accurately analyze and predict future system behavior without precisely knowing the forward physical model. ML methods generally have a more powerful capacity for FDP without the assumption of data distribution, smoother and more intelligent FDP processes with fewer processing stages and less human intervention, and, moreover, less prior-knowledge requirements for more complex components or systems to be modeled [6].

Consequently, data-driven ML methods have long been applied in various industrial FDP applications. A typical ML pipeline generally consists of three steps [7], i.e., data preprocessing, feature extraction and classification or regression. The performance of ML heavily depends on the manually predefined feature extraction rules. In the past decade, with the great development of mega-scale open datasets [8], evolutional computing capacity of new GPU architectures [9] and innovative neural network training methods [10], deep learning [11] can hierarchically extract highly-abstract features in an end-to-end way from the labeled training dataset. Due to its superior performance over ML methods, deep learning (DL) has gained remarkable success in the tasks of computer vision, natural-language processing, etc. In the community of industrial FDP, researchers have also made great efforts to introduce DL techniques into different and unique industrial FDP scenarios, and tremendous progress has been witnessed.

At present in the era of Industry 4.0 [12], the emerging of Big Data [1,13], Internet of Things (IoT) [14,15], and artificial intelligence (AI) technology [16,17] are now promoting the transformation of PHM (specifically FDP in this paper) from traditional single-sensor-oriented diagnosis to system-wise intelligent diagnosis and prognosis. When the traditional physical model-based PHM technology is progressing slowly in the face of unprecedented complex systems, the scientific “The Fourth Paradigm” [18] based on Big Data collected from IoT and supported by modern AI technology is also making industrial systems truly intelligent.

### 1.2. A Survey of Relevant Reviews

To summarize the current research of intelligent FDP, there are a number of outstanding surveys on the topic of intelligent FDP [1,7,19,20,21,22,23,24,25,26,27,28]. They conduct extensive review on existing literature quantitatively and qualitatively from their unique viewpoints, and identify the trends and ideas of FDP methods for different scenarios.

Xu et al. [1] analyzed existing issues and challenges in the Big Data era from different driving factors, such as data quality and cost balance, method selection, application problems, and deep utilization. Li et al. [19] summarized the common fault types of sensors in monitoring and control systems and presented the latest fault diagnosis methods that combined different advanced technologies. Furthermore, Tang et al. [27] reviewed the DL applications toward fault diagnosis methods for rotating machinery according to its major components, including bearing, gear, and pumps. A comprehensive review of Big Data-driven intelligent FDP for mechanical systems was given by Lei et al. [28], wherein the latest cutting-edge research results are focused, e.g., deep transfer learning-based FD, Big Data-driven RUL prediction, data-model fusion prognosis, etc. In addition, Fernandes et al. [20] provided a systematic literature review of ML methods for mechanical FDP in manufacturing. They examined and characterized the research in more details based on five basic research questions.

### 1.3. Motivation

The aforementioned review work provides a very good foundation for the work in this paper. Some surveys concentrate on FDP for specific type of device, e.g., machinery [20,21,22,23,24,27,28], wind power converter [25], lithium-ion battery system [26], while some focus on specific FDP method, e.g., deep domain adaptation [21], attention mechanism [22], recurrent neural network (RNN) [23], etc. Most of these reviews cover the data-driven ML techniques, but few of them give a comprehensive overview of the generic DL techniques used for industrial FDP. Moreover, due to the rapid development and iteration of DL techniques in recent years, a large number of excellent DL architectures and algorithms have emerged, bringing new opportunities to the promotion of FDP. The most up-to-date trends of recent a couple of years in industrial FDP, especially about emerging DL architectures, as well as the future trends in the next few years, are rarely covered by relevant reviews. To the best of our knowledge, there is currently no review paper of the Transformer technique’s application in intelligent FDP.

Therefore, a review to comprehensively cover the latest development of DL techniques for intelligent industrial FDP is still left blank but desired. In order to track the latest achievement of DL techniques for intelligent industrial FDP, we conduct a comprehensive survey on relevant literature of the past 5 years in this paper. The main contributions of this paper are as follows:

1. From a different viewpoint of data analysis, we provide a generalized definition and mathematical formulations for FDP problems compared to previous work.
2. We collect and summarize recent advances of recent 5 years for intelligent industrial FDP, review and analyze them from the perspective of DL techniques.
3. The emerging DL architectures, including generative adversarial network, and transformer and graph neural network, are investigated in the survey to provide an up-to-date view of the latest research trends of intelligent FDP.
4. Challenges encountered in current research are discussed from the aspects of data imbalance, compound faults, multimodal fusion and edge implementation, which are seldom analyzed by other literature. Possible solutions are also provided.

The rest of this paper is organized as follows. Section 2 gives the problem formulations. In Section 3, we elaborates the FDP methods of emerging DL techniques. Its detailed analyses are given in the followed Section 4 and Section 5. In Section 6, the major problems encountered in the current research are summarized and the trend is prospected. The conclusions are finally drawn in Section 7.

## 2. Problem Formulation

Different from previous work that deals with specific industrial faults and analyzes them from the aspect of physical model or fault mechanism, we analyze the problem of FDP from a novel viewpoint of data analysis. In this section, we give the generalized definitions of faults and the mathematical formulations of FDP problems.

### 2.1. Definitions of Faults

In general, the condition monitoring results of certain object in industrial systems experiences changes all the time, and not all changes in sensory data are failures or faults. Here are some common senses:

- Changes caused by random noise are not necessarily faults, but when the variance of the noise changes, it is generally considered to be a fault.
- Fluctuation within a stable range in a certain operation condition is not a malfunction. In different operating conditions, this fluctuation may be different.
- A change that breaks the current pattern is a fault.

**Figure 1:**
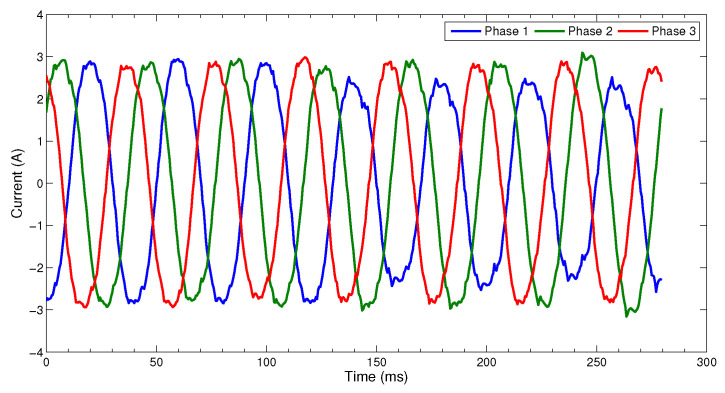
An example of three-phase current waveform.

Figure 1 gives a comparison of the normal three-phase current waveform and the current waveform of interturn short-circuit fault under the same working condition. At no point does the current amplitude exceeds the working condition mode range, but the (blue) curve pattern of t>125 ms changes and it is a fault. Therefore, we consider that the core part of FDP is to discriminate the faulty patterns from normal working patterns which are represented in sensory data, and to build a health index that indicates the changing trend in working patterns.

### 2.2. Mathematical Formulations of Fault Diagnosis

Given *N* physical variables (such as pressure, current, temperature) within a specific time range $T=[t_{1},t_{2}]$ measured by a number of sensors (such as strain gauges, Hall sensors, temperature sensors, etc.) at a specific position of a specific device, we set $M\left(T\right)=\{m_{i}\left(T\right)|i=1,2,\cdots ,N\}$. When the current operating condition is *p*, the fault indicator function $f_{\theta }\left(M\left(T\right),p\right)$ is to judge whether the current state *s* as in Equation (1) is normal or not, its value range of $f_{\theta }$ is {0,1}, and θ is the parameter of *f*.

(1) $$s=f_{\theta }\left(M\left(T\right),p\right)$$

when the monitoring variable M(T) and the working mode *p* are known, the corresponding fault state is also determined theoretically, i.e., for a certain type of device, its fault indicator function *f* is determined.

In this way, the problem of fault diagnosis becomes the process of solving the parameter θ of the fault indicator function *f*. The determination of function parameters θ can be explicitly solved by forward modeling of physical models, but it is often too complicated or even unsolvable. The data-driven fault diagnosis methods make use of the existing data, and tries to mine the parameter θ of *f* backward from the data [7]. It then becomes the following problem as in Equation (2), that is, searching for a certain point $\theta^{\prime }$ in the parameter space Θ, so that its output pattern on a large number of data samples is the least different from the real situation, thereby turning it into an optimization problem:

(2) $$\arg\min_{\theta^{\prime }\in \Theta }∥s^{\prime }-f_{\theta^{\prime }}\left(M^{\prime }\left(T\right),p^{\prime }\right)∥.$$

Among them, $s^{\prime }$ and $\left(M^{\prime }\left(T\right),p^{\prime }\right)$ are the labels and data vectors in the known sample set.

If the current device status is judged as fault, the fault can then be classified. The current pattern is compared with the fault patterns in the fault database, the smallest deviation degree between the current fault and each fault pattern can be searched. It is worth noting that since the original data M(T) used for diagnosis is usually high dimensional and redundant in feature spaces, it is usually necessary to perform feature selection, feature extraction or feature fusion on the original data to reduce the data dimension.

### 2.3. Mathematical Formulations of Fault Prognosis

**Figure 2:**
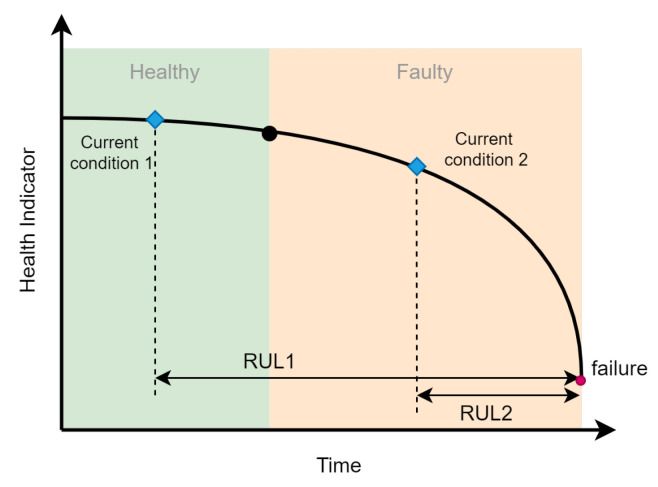
Schematic diagram of life cycle.

One major challenging problem in fault prognosis is the remaining useful life (RUL) estimation of the device whose specific meaning is shown in Figure 2. It is necessary to select an appropriate health indicator for RUL estimation, which can well reflect the change in the degradation degree of device health, and there is a corresponding threshold to indicate when will the device reach a functional failure.

Given *k* known historical data and their corresponding health feature sequence $\left\{f_{i}\left(n\right)|i=1,2,\cdots ,k,n=1,2,\cdots ,N\right\}$, where *N* is the length of the known health feature sequence, the dataset $\left\{T_{i}\left(l\right),f_{i}\left(l\right)\right\}$ can be formed according to all the historical data and the corresponding sequence of health indices. According to the determined device-life degradation model *g*, we can perform fitting via regression on $\left\{T_{i}\left(l\right),f_{i}\left(l\right)\right\}$ to determine the model parameters of the degradation model *g*. Given the current observation data health indicator sequence f(n), the degradation model *g* is used to extrapolation predict and estimate the evolution trend $\hat{f}$ of the predicted features. The estimated evolution curve $\hat{f}$ obtained is then compared with the failure threshold. When $\hat{f}$ exceeds the failure threshold for the first time at time $T_{f}$, the device fails. Assuming that $T_{N}$ is the time length of known observation data, RUL of the device is

(3) $$RUL=T_{f}-T_{N}.$$

The key point of fault prognosis is the choice of degradation model. The factors considered include the global degradation mode, short-term degradation characteristics, the amount of data available for modeling and the data noise level, etc.

## 3. Modern Deep Learning Techniques for Intelligent Industrial FDP

### 3.1. Modern Deep Learning Techniques

As a young and developing field of AI, ML techniques try to discover knowledge from a large amount of historical data for prediction or classification on new data. More specifically, it is designed to find a projection to fit the input data for desired results, which is often too complex to be explicitly formulated. In terms of application purposes, supervised machine learning is mainly divided into two categories [29]: classification and regression. The former learns the boundaries between categories to achieve classification of new data [30]. The latter fits regularities to the data to predict the properties of new data points. Correspondingly, fault diagnosis is actually a classification problem, and fault prognosis is a regression problem.

As a subset of ML, the emerging DL is currently the hottest topic in AI. It is originated from the paper [10] published in 2006 by Hinton et al. This paper reveals two characteristics of deep learning. The first is that the neural network with multiple hidden layers has excellent potential for learning more representative features from raw data which are generally designed manually in traditional ML methods. The second is that the difficulty of training deep neural networks can be overcome by layer-by-layer pre-training using the method of unsupervised learning in the Restricted Boltzmann Machine (RBM).

The concept “deep” in deep learning is compared to traditional machine learning algorithms, such as SVM, ANN, and other shallow learning methods, in which there are more layers of non-linear functions in deep learning methods. In traditional shallow neural learning methods, data sample features need to be manually extracted. Conversely, DL automatically learns to obtain feature representations by performing layer-by-layer feature transformation on original data via back-propagation, and these hierarchical feature representations are highly abstract and task-oriented. One of its major merits is that it can complete the learning in an end-to-end way directly from raw data to results of classification and regression tasks.

Typical DL architectures include deep belief network (DBN) [31], autoencoder (AE) [32], convolutional neural network (CNN) [33], and RNN [34]. With the rapid development of DL techniques in these years, many new architectures have been proposed and introduced into the tasks of intelligent industrial FDP. Examples are generative adversarial network (GAN) [35], transformer [36], and graph neural network (GNN) [37]. Similarly, CNN is prospering again, due to the progress made in the fields of computer vision in recent years.

### 3.2. Categorization and Literature Trends of DL Techniques for Industrial FDP

**Figure 3:**
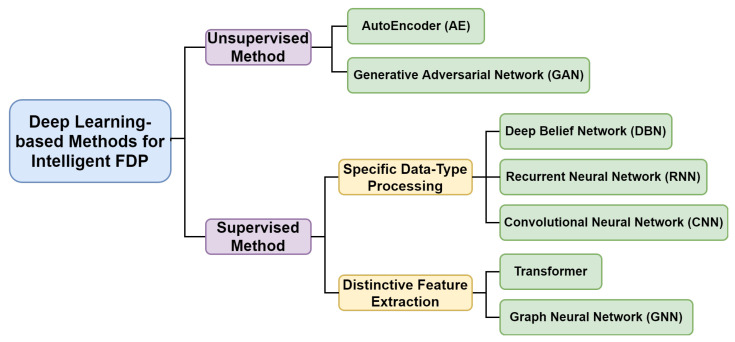
The categorization of deep learning techniques in intelligent FDP.

Figure 3 shows the categorization of major DL-based approaches used in intelligent FDP. According to the supervision type, they can be divided into unsupervised methods and supervised methods. The former tries to find the inherent common pattern within data which are unlabeled, while the latter refers to methods that learn highly non-linear relationship between the input data and its paired labeled output. More specifically, the supervised methods can be further divided into processing of specific data types or extraction of distinctive features, depending on their objectives. Their detailed introductions will be expanded in the following sections.

**Figure 4:**
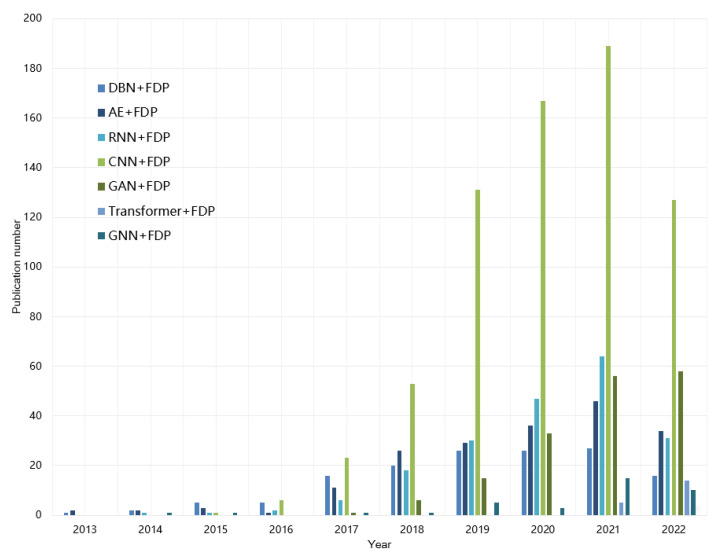
Publication trends of deep learning methods in intelligent industrial FDP.

Figure 4 illustrates the number of journal publications of deep learning methods in intelligent FDP from January 2013 to September 2022 on Web of Knowledge. As can be seen, the number of papers published is increasing year by year, and CNN-based FDP methods account for the majority of all methods. The publication number of typical DL architectures, such as DBN and AE, are stable or growing with relatively slower speed. Note that emerging network architectures are also gradually attracting the attention of researchers.

## 4. Part I: Unsupervised DL Methods for Intelligent Industrial FDP

Unsupervised DL methods are not fed with labeled information, so it is necessary for them to mine the inherent structure and pattern within data. Unsupervised DL methods generally does not solve the tasks of FDP in a direct way, but also serve for peripheral tasks that are also crucial, such as feature reduction and data generation.

### 4.1. Autoencoder (AE) for High-Dimensional Feature Reduction

**Figure 5:**
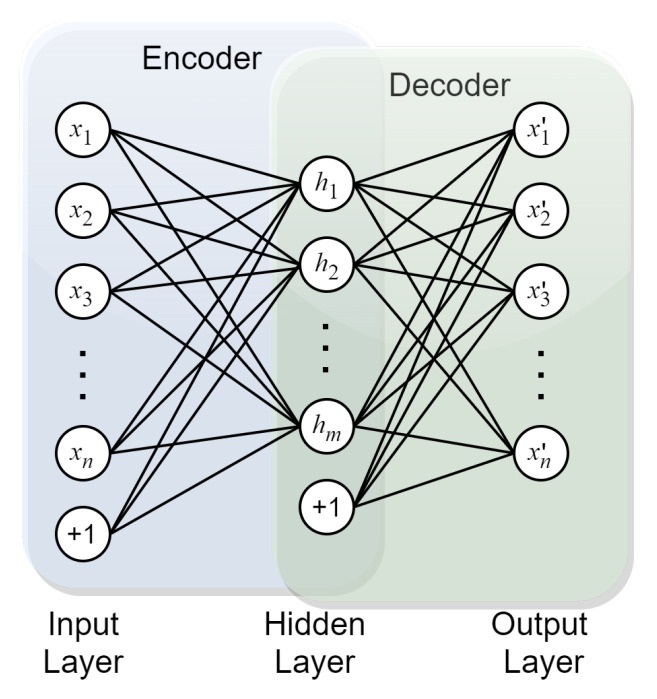
Basic structure of AE.

Autoencoder (AE) is an unsupervised architecture which assumes that the output being encoded and decoded is the same with the input. In this sense, the encoder part can be used for feature reduction where high-dimensional input data can be converted into low-dimensional encoded vectors. The idea of an encoder–decoder is also widely adopted by other DL architectures such as CNNs. A simple architecture of AE is illustrated in Figure 5. AE can also be divided into standard AE [38,39,40], denoising AE [41], sparse AE [42], variational AE [43] and contractive AE [44], etc.

AEs have been widely used for feature extraction and fault classification, and have demonstrated powerful feature extraction and non-linear dimensionality-reduction capabilities and robustness in practical FDP applications. In [45], a sparse AE is designed to automatically extract degradation indicators for followed fault detection in multi-component system. Ref. [46] use multi-layer sparse AE as a multi-sensor feature fusion and extraction method combined with DBN for bearing fault diagnosis. A list of recent publications of AE-based intelligent FDP are given in Table 1. As seen, in order to obtain better performance, stacked AEs are preferred to be used in different scenarios, while the borders between different types of AEs are breaking down and leading to fused architectures, e.g., sparse denoising AE. Despite the above advantages, it still suffer from the situation that meaningful features sometimes cannot be easily extracted due to the inherent properties of AEs. Moreover, its capability is generally highly correlated to its training samples.

### 4.2. Generative Adversarial Network (GAN) for Data Generation

**Figure 6:**
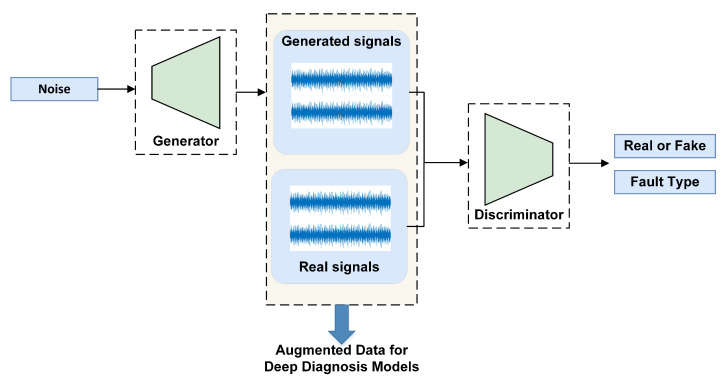
A basic example of data augmentation using GAN.

An important requisition for supervised deep learning methods is the massive amount of training samples. However, in many practical scenarios, training data collected at hand are scarce and imbalanced, which is reflected on the ratio of numbers of positive and negative samples, as well as the known fault patterns. It is a well-known problem of small sample or small data. Traditional over-sampling techniques can hardly capture the data distribution and will easily lead to over-fitting [49]. Firstly, succeed in computer vision from 2014 by Goodfellow, generative adversarial network (GAN) [35] is an unsupervised method that is able to generate realistic samples via a minimax game between two networks. It consists of a generator network to generate samples and a discriminator network to judge the likeness of the generated samples. The generated realistic fake data fit within the distribution of the training data, which outperforms the traditional over-sampling methods, such as synthetic minority oversampling technique (SMOTE) [50], by a large margin. As a result, GAN has shown outstanding performance in many areas beyond computer vision. In the field of FDP, GANs have gradually been adopted, and it has show promising results compared with other architectures. The basic idea of data augmentation using GAN is illustrated in Figure 6.

Initially, GAN is mainly adopted for normal or faulty sample generation, either for images or for signals. Figure 6 is an example GAN for data augmentation for the training of deep fault diagnosis models. Usually, the capacity of modeling data distribution in GAN can be further extended for fault diagnosis. For example, the trained generator can be used to fix a faulty sample, and the fault can then be located by sample comparison [51]. Moreover, this adversarial learning strategy of GAN has also been widely implemented to tackle the problem of domain shift of data distribution for fault diagnosis under different working conditions or environments, i.e., the distribution of available training data in the source domain is different from that of data to be tested in the target domain, making the trained model hard to be generalized [52]. It is a very challenging issue usually faced by industrial applications.

Due to its special and excellent property, GAN has, consequentially, received significant attention when dealing with intelligent FDP of real industrial systems. A list of recent methods based on GANs are given in Table 2 for more comprehensive and detailed information. The current work mainly focuses on the gaming strategy of GAN to achieve the goal of more realistic sample generation and cross domain adaption for intelligent FDP. Ref. [49] set up an infoGAN-based failure-prediction algorithm, and it uses an auxiliary GAN to enforce consistency of the generated samples and their corresponding labels. Ref. [53] propose to use deep feature enhanced GAN to ensure the accuracy and diversity of synthesize samples, thereby improving the performance of rolling bearing imbalanced fault diagnosis. Aiming at the problem that in real industries only data in machine healthy condition can be collected in advance, literature [54] propose a multilabel 1-D GAN to generate damage data of industry equipment, and the fault diagnosis accuracy was improved with these generated data. Ref. [55] jointly use labeled samples in auxiliary domain and unlabeled samples in target domain via domain-adversarial training in order to enhance the adaptability of samples in auxiliary domain to target domain and improve the transfer performance.

Despite the fact that GANs can generate samples with the same distribution, it is still difficult to judge or evaluate the quality of generated 1-D signals, as opposed to the image generation. Moreover, how to ensure that the adversarial training process converges to the desired destination is also a challenge. Lastly, as faulty sample generation is always on the top of the objective list, the way of combining prior knowledge from experts to improve the generation is also an important issue to be explored for real industrial applications.

## 5. Part II: Supervised DL Methods for Intelligent Industrial FDP

Different from the unsupervised learning way that does not utilize labeled input data, supervised learning methods use a training set with inputs and correct outputs to teach models to yield the desired output. For intelligent FDP, supervised learning methods can be used to extract distinctive features for the specific task from specific types of sensory data.

### 5.1. Deep Belief Network (DBN) for Fault Features Mining

**Figure 7:**
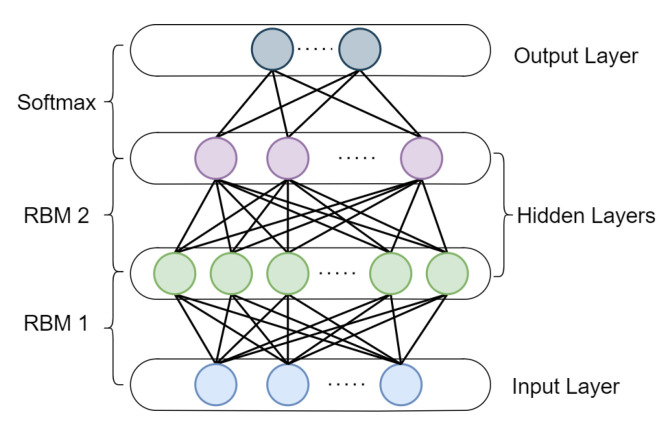
Basic structure of DBN.

The traditional neural network is more computationally efficient when it has only few hidden layers, so it is mostly used to solve some relatively simple mapping modeling problems. DBN is a network constructed by stacking RBM which is a special type of generative stochastic neural network, including visible units and hidden units, and a basic example of DBN with two hidden layers is shown in Figure 7. It can be trained through pre-training the stacked RBMs. Based on DBN with multiple hidden layers, it can remove the dependence on prior-knowledge and adaptively extract fault features for diagnosis. It is also able to process non-linear high-dimensional data, thereby effectively avoiding problems, such as dimensional disaster. Therefore, DBNs are well suited for dealing with fault diagnosis of industrial Big Data.

Until now, plenty DBN-based researches have been carried out in this area, and widely used in fault diagnosis of aircraft engines [66], reciprocating compressors [67,68], gearboxes [69,70,71,72], rolling bearings [73,74,75,76], power transformers [77,78], etc. Current studies generally either use DBN as a classifier in a supervised way, or replace traditional signal processing methods to mine fault features in an unsupervised way. A compilation of recent work on DBNs for intelligent FDP are given in Table 3 from the classification of five aspects, along with their objects.

As a very classical technique in DL, DBN maintains a great deal of parameters to be set, and once inappropriately handled, it will affect its generalization and limit the accuracy, especially compared with other modern DL techniques. As a result, DBN is now being widely combined with other architectures, e.g., CNN, to achieve better performance, which can also been observed in Table 3.

### 5.2. Recurrent Neural Network (RNN) for Time-Series Data Processing

**Figure 8:**
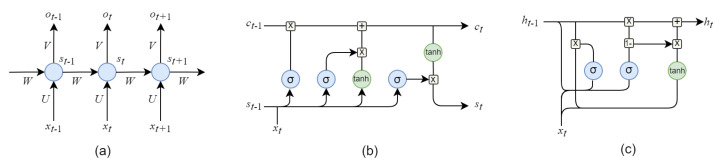
Unit comparison of (**a**) basic RNN, (**b**) LSTM, and (**c**) GRU.

Compared with other architectures, recurrent neural network (RNN) [34] assumes that the input and output are not independent of each other, i.e., it tries to learn long-term dependencies from sequential or time-series input data. RNN contains non-linear recurrent units with directed cycles, combined with unit hidden states, so that time-series information can be preserved. Due to this structure, the state of the hidden layer is not only affected by the input data, but also by the previous calculation results, showing better dynamic characteristics. RNN is theoretically an ideal non-linear time-series forecasting tool and a universal approximator for dynamic systems. Common RNNs include gated recurrent unit (GRU) [87,88] and long short-term memory networks (LSTM) [89,90,91], which have become one of the most effective FDP methods for time-series data at present. Their basic unit comparison of them are given in Figure 8.

Since long-term condition monitoring data are collected, RNN-based methods are in great demand in intelligent FDP. Ref. [91] proposes a convolutional LSTM that simultaneously extracts time-frequency domain features and models their long-term dependencies of vibration signals from bearing. The work in [92] utilized LSTM for fault diagnosis and RUL estimation on time-series aeroengine data. Ref. [93] use a RNN to implement early warning in the fault creep period for nuclear power machinery, together with principal component analysis, wavelet analysis, and Bayesian inference model. Ref. [34] design a fault prognosis approach with the degradation sequence of equipment based on LSTM, which uses the concatenated feature and operation state indicator for RUL estimation. Some of recent methods based on RNNs are listed in Table 4 according to their RNN types and purposes, e.g., fault diagnosis and RUL estimation.

On one hand, the special structure of recurrent units with directed cycles enable RNN to better modeling time-series information and on the other hand, it makes that the training of RNN is generally much slower than that of other architectures such as CNNs, which poses a great computational requirement for industrial computing centers. Meanwhile, similar to CNN, RNN is also sensitive to training data, and when the fault feature is weak or distorted by noise, it is also hard to maintain good performance.

### 5.3. Convolutional Neural Network (CNN) for Image Fault Diagnosis

The convolutional neural network (CNN) is inspired by biological visual perception mechanism. It has unique structural characteristics, such as local connection, weight sharing, and pooling, which enables CNN with strong feature learning and representation ability. At present, CNN are mainly used in fault diagnosis, and it can hardly realize the status trends analysis of equipment or fault prognosis. In the field of intelligent FDP, there are generally three situations. A list of recent publications on intelligent FDP based on CNN architectures are given in Table 5. Details are described in the following subsections.

#### 5.3.1. The Monitoring Sensors Are Cameras

When the device fault can be captured by camera, i.e., there are evidences reflected at pixel level, the CNN-based methods can obtain better diagnosis results, such as in the fields of machinery and circuits. Ref. [101] proposes a fault diagnosis strategy for rotating machinery based on CNN using infrared thermal images. Ref. [116] integrates an attention mechanism into CNN to efficiently extract the fault features of analog circuit. Similarly, Ref. [117] use a encoder–decoder-like CNN to find cracks on device surface in complex background. The diagnosis of such image data generally can hardly achieve precise quantitative description of the faults, it can usually only obtain the qualitative trend of the device faults.

#### 5.3.2. Conversion from Other Sensory Data into Images

**Figure 9:**
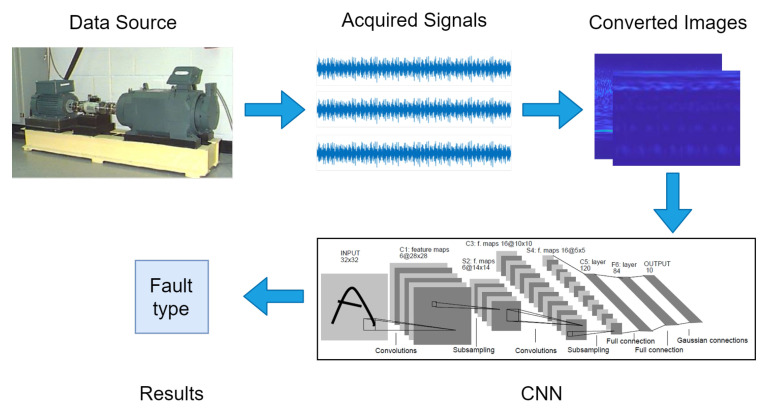
A typical fault diagnosis pipeline based on signal-to-image conversion and CNN.

Usually the monitoring variable observed by the sensor is a one-dimensional signal, which is different from a two-dimensional image. In order to leverage the powerful feature learning ability of CNN, many researchers consider converting one-dimensional signals into two-dimensional images, and then input them into CNN for classification or recognition. For example, Ref. [104] propose an intelligent fault diagnosis method for aeroengine sensors combining a CNN with time-frequency analysis wherein the signal recognition problem is transformed into an image-recognition problem. An example pipeline is illustrated in Figure 9. Many of these work puts their main focus on how to convert to two-dimensional images. Common methods include wavelet transform [102,104,108], S-transform [118], phase space reconstruction [119], etc. These two-dimensional time-frequency distribution images generated by transformation often have simpler backgrounds than natural images. The quality of these transformation methods directly affects the performance of CNN. If there is little difference between the two-dimensional images of fault and non-fault signals, the accuracy of CNN classification will also be unsatisfactory.

#### 5.3.3. 1-D CNN for Signal Processing

Actually, two-dimensional convolution operations can also be decomposed two one-dimensional convolutions vertically and horizontally. Therefore, another attempt direction is that tries to fit two-dimensional CNN to one-dimensional data, i.e., 1-D CNN [54,113], which is specialized for temporal signals [120]. This operation is inherently suitable for sensory data, and has been widely used for intelligent FDP in recent years. For example, Ref. [121] presents a 1-D CNN-based approach to automatically learn features for rub-impact fault diagnosis from the raw vibration signals of a rotor system, and [114] establish a fault identification model based on the powerful feature extraction and complex data analysis abilities of 1D-CNN. Due to its inherent properties, many modern techniques for 2-D CNN can be imported into 1-D CNN for better signal feature extraction, such as attention [112], lightweight design [122], and dilated convolution [123].

Although CNN has provided an alternative way to process different types of condition monitoring data, there are still limitations. Firstly, the conversion from signal data to image is equivalent to the quantization process of imaging, which means that important details of signal intensity can be naturally omitted when projecting to pixel bins. In this way, subtle abnormality in the early stages can easily be ignored by convolution and pooling operations. Lastly, the methods for conversion should also been carefully designed to prevent overfitting. Furthermore, it is also a challenge for CNN-based FDP methods to achieve real-time diagnosis since they are with relatively high computational overheads for image data.

### 5.4. Transformer for Self-Attention Feature Extraction

Initially designed in natural-language processing, attention mechanism is a technique that can model sequence dependencies, which allow a model to focus only on a set of elements and to decompose a problem into a sequence of attention-based reasoning tasks [124,125]. The attention mechanism now has been adopted in various deep learning architectures, such as CNNs and RNNs. Transformer architecture [126] abandons all the recurrent and convolutional structures, and only contains multi-head self-attention (MSA), multi-layer perceptron (MLP), and a basic fully connected layer [127] to capture the long-term dependencies between elements in a sequence without considering their distance, which can consider the global information comprehensively.

**Figure 10:**
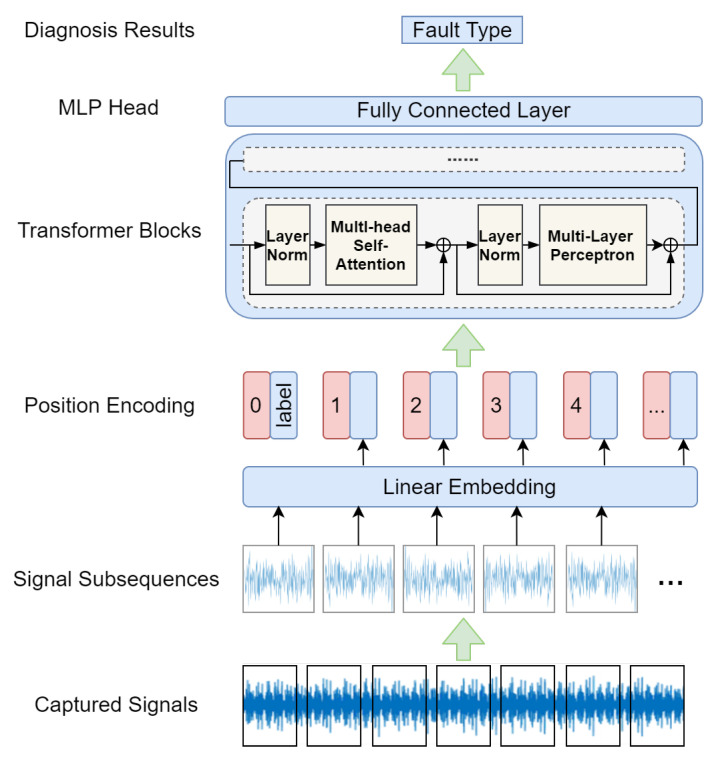
An example pipeline of transformer-based fault diagnosis.

In Figure 10, we illustrate an example of fault diagnosis pipeline using transformer. The captured signals are firstly cropped into signal subsequences according to their original positions, which is then mapped into a high-dimensional vector through linear embedding and followed by trainable position encoding to retain the position information of the signal. Vectors are then fed into multiple stacked transformer blocks for long-distance modeling through layer-normalized MSAs and MLPs. Finally, the extracted features are input into the MLP head, i.e., fully-connected layer, for the classification results. Common loss functions for other classification tasks are also used.

Due to the outstanding global information modeling ability, transformer has outperformed other architectures in feature extraction for many tasks, and is a hot research topic of FDP in these two years. Ref. [127] proposes a time-series transformer which utilizes raw vibration signals for the rotating machinery fault diagnosis, and it tries to capture translation invariance and long-term dependencies with a new time-series tokenizer. Different from [127], Ref. [128] designs a time-frequency transformer with a fresh tokenizer and encoder module to extract effective abstractions from the time–frequency representation of vibration signals. Ref. [36] use an integrated vision transformer (ViT) based on the soft voting fusion method to diagnose the bearing fault with high accuracy and generalization. For RUL prediction, Ref. [129] propose a transformer-based encoder–decoder structure with a dual-aspect encoders design to extract features from the sensor and time step simultaneously, while adaptively learning to focus on more important part of input and processing long data sequences.

Some recent work of these two years for intelligent FDP based on a transformer are given in Table 6. As can be observed, transformer-based FDP methods are gradually being used as excellent feature extractors and for time-series data processing, due to their outstanding performance in modeling long-distance information in input data, compared with CNNs and RNNs.

Owing to the ability of long-range modeling of data, it side-effect is that its local information modeling ability is relatively lower than CNNs and RNNs, and there are also attempts to make up the shortcoming through combining transformer with CNN or RNN. The second limitation is its computational efficiency because of its special structure, and it is undoubtedly the current hot spot for DL community. However, then again, there is still much to be further explored on this topic.

### 5.5. Graph Neural Network (GNN) for Relationship Modeling

Although the above deep learning techniques can effectively capture the hidden features or model the inherent knowledge from input data in an end-to-end way, most of them ignore the inter-dependencies between data or various physical measurements of multiple sensors [140]. Since [141] first applied neural networks to directed acyclic graphs, graph neural networks (GNN) have successfully handled data characterized by complex spatiotemporal relationships [142]. Although deep learning effectively captures the hidden patterns in Euclidean domains, more data are generated from non-Euclidean domains and represented as graphs with complex spatiotemporal relationships among objects. GNN tries to model the relationships with graph representations, i.e., feature node and adjacency edge, and concentrate on the tasks of node classification (node level), edge classification and link prediction (edge level), and graph classification (graph level) [140,143]. GNN can be integrated with other architectures and extended to graph convolutional networks (GCNs) [144], graph attention networks (GATs) [145], graph autoencoders (GAEs) [146], etc.

A graph structure in GNN can be generally represented by a node feature matrix, an adjacency matrix and a set of weighted edges. It can propagate the node information through the edges of a graph via graph operations, such as graph convolutions, and learn a promising node or graph representations. The most commonly used GNN is GCN, and many operations in GCN can find their similar counterparts in CNN, such as convolutions on nodes to aggregate the information of connected neighbor nodes along the weighted edges, Relu function for non-linear activation and pooling layer to reduce dimensions, though there are very small differences in operations in practice.

**Figure 11:**
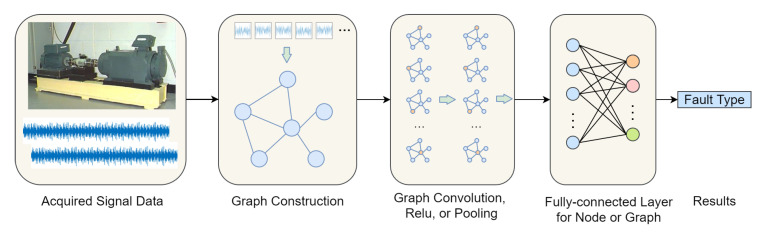
An example pipeline of fault diagnosis using GCN.

Owing to the capability to model relationships in data, GNN has been receiving attentions from researchers in the FDP community recently, and the challenges faced in FDP are the appropriate way of constructing and realizing the graph [142]. Figure 11 gives an example diagnosis pipeline based on GCN. Similarly, [144] present a GCN-based fault diagnosis method that uses a association graph constructed from prediagnostic results and adjust the graph via using a hybrid of measurements and prior knowledge, which obtained good diagnosis results. When dealing with time-series data, the work in [140] constructs three kinds of graphs for fault diagnosis and prognosis according to the time-series subsample types as univariate and multivariate data, respectively. Ref. [147] proposes an interaction-aware GNN for fault diagnosis of complex industrial process, which transforms sensor signals into a heterogeneous graph with multiple edge types and employ a GNN to extract fault feature of one edge type, so it can learn implicit interactions between sensor signals.

In Table 7, more recent GNN-based intelligent FDP methods are listed for the references of readers. It can be observed that GNNs has a high popularity in the last two years. On the basis of knowledge graph, GNN is recognized to reason or infer knowledge, which realizes the promotion from perception to cognition of AI. As a result, at current stage of research, the explicit incorporation of (prior) knowledge for constructing graphs in GNN instead of currently using a large amount of training data, and more generalized knowledge inference are desired and beneficial for FDP. GNN is expected to show greater potential in subsequent studies for intelligent industrial FDP.

## 6. Challenges and Possible Solutions

This paper has provided a systematic literature review of deep learning based intelligent industrial FDP. It can be concluded that there are a lot of interest in using CNN, DBN, or RNN for fault diagnosis purposes, but when architectures develop, more complicated but powerful methods have been introduced into FDP. GNN, Transformer, and GAN are gradually receiving attention and their performance has also begun to surpass traditional methods. Although the deep learning methods have been applied in the intelligent FDP of industrial systems, there are still several challenges that need to be explored and solved. In this section, we analyze the open challenges from the four aspects of data imbalance, compound fault type, multimodal data fusion, and edge device implementation, and provide possible solutions.

### 6.1. Imbalance Problem in Industrial Applications

In practical industrial applications, the acquisition of typical data (including historical health data, fault data, and simulation data) of some devices is usually expensive, labor-intensive, and sometimes impossible [156]. Even if the state data of the system can be acquired, it often has strong uncertainty and incompleteness, these problems increase the difficulty of FDP. At present, the total amount of existing data can only support the implementation of traditional methods or shallow machine learning methods. It is still a challenge to train robust intelligent FDP models with limited data and works well under complex working conditions. The second problem [157] is the imbalance data that (1) there are too few fault samples and too much duplicated normal data samples; and (2) there is an open set of fault modes that many of the modes may not be encountered in operation.

One possible way is to run long-term laboratory tests or simulation for every single device and the whole system, in order to simulate various working conditions in the laboratory, and find all possible fault modes of devices and the system. However, obtaining complete fault data of the entire system sometimes is expensive and infeasible [156]. In terms of intelligent FDP techniques, it could be solved from the following aspects.

#### 6.1.1. Task-Level Transfer Learning

Despite the imbalance in local systems, there are a large number of similar devices or subsystems in other industrial, mechanical, power grid systems, etc. These devices and subsystems share the similar architecture or composition, and they have accumulated a certain amount of historical health data. The utilization of these large amounts of useful data or knowledge from other systems for the FDP of local industrial system, i.e., task-level transfer learning, becomes an efficient and promising approach. It emphasizes the transformation data, feature, knowledge or model to different fields. At present, transfer learning-based methods have been implemented in other fields such as image recognition, and several pioneering work has been completed for intelligent FDP. Ref. [158] adopt the knowledge transfer scheme and use a multi-input multi-output convolutional network to extract domain-invariant feature representations and classifiers from the labeled dataset from scientific test rigs and the unlabeled dataset from industrial application to be tested.

#### 6.1.2. Data-Level Augmentation

One direct way is to generate more balanced/diverse data to enhance the training sets for FDP models. Traditional data augmentation through transformations, such as translation, deformation, and scaling, has low computational cost and is easy to implement, which is a simple and efficient way to generate a large amount of labeled samples to improve FDP performance with limited data. However, the generated samples can be considered as local distortions of existing labeled sample points in high-dimensional space, i.e., they are still with limited diversity. GANs offer a good option to generate more realistic or vivid data samples with the same original data distribution of minor fault patterns for both 2-D image data and 1-D timer-series signal data, as we have analyzed in Section 4.2.

#### 6.1.3. Model-Level Meta-Learning

Meta-learning is a flexible framework which can learn to obtain the ability of extracting meta-knowledge from multiple relevant tasks to gain generalization on various tasks, in order to guide the learning and improve its performance on target tasks without training from scratch [159,160]. Currently, the studies of model-level meta-learning for intelligent FDP with imbalanced data are still in their earlier stages. Some work [159], mostly based on metric-based meta-learning, has explored its implementation in industrial FDP, and shown excellent accuracy and robustness on public datasets. However, it needs further development and verification in operational industrial systems.

### 6.2. Lifting Diagnosis from Single Faults to Compound Faults

Most of the modern deep learning-based intelligent FDP methods are only applied in the single-fault diagnosis. However, in actual complex industrial systems, several kinds of single faults may exist simultaneously, which means several components or devices may break down together, resulting in compound-fault modes [103]. Usually, these faults are related to each other and affect each other at the same time. The signals captured by sensors may be coupled with multiple fault signals, and the generic FDP methods that work for one single fault will inevitably fail in compound-fault modes. In addition, the compound-fault samples are also difficult to collect and label, which further limits the application of the existing deep learning-based methods [161]. In operational complex industrial systems, compound faults are generally more dangerous and harmful than a single fault [162]. It has, therefore, become a key issue to be solved for complex industrial systems.

Traditional compound-fault-diagnosis methods rely heavily on either prior knowledge inference or signal analysis [161], which is difficult to be applied in operational industrial systems. Identifying and decoupling the compound fault are still a great challenge for intelligent FDP. The effective separation of fault characteristic components is the core of compound-fault diagnosis [163]. Ref. [103] uses a multi-label CNN to achieve compound fault diagnosis based on the 2-D time-frequency features in an end-to-end way. Ref. [164] propose a deep ensemble capsule network that combines multiple decoupling capsule network individually trained on one sensory data in a way of ensemble learning to effectively decouple the compound fault into individual faults. In [162], a decoupling classifier is designed to decouple the compound fault into single faults by outputting multiple labels for samples.

Considering that the compound-fault-sample data are always scarce, it is also important to use the single fault data to train the compound-fault decoupling model with the help of the knowledge learned from the single fault mode data. The decoupling classifier in [162] is trained on a dataset only containing normal and single fault samples. To address the problem of identifying unknown compound faults, Ref. [161] present a zero-shot learning model which classifies the compound faults according to the similarity measure between the signal features and the semantic features of the compound faults to identify the categories of unknown compound faults. Actually, the scarce of compound fault samples is a key issue to improve the practicability of the intelligent compound-fault-diagnosis methods.

### 6.3. Boosting Intelligent FDP with Multimodal Fusion

On one hand, an individual sensor can hardly provide the complementary and thorough information of complex industrial devices, and various signal transfer paths from the fault point to the location of sensor, so it is necessary to place several sensors at different places to capture more comprehensive and accurate information for the faults [164]. Therefore, in industrial systems, there are always multisensory data used for intelligent FDP. In recent years, intelligent FDP based on the fusion of multi-source homogeneous information has been thoroughly explored and discussed. On the other hand, a fault can be reflected in several relevant sensors with heterogeneous platforms simultaneously, such as current, voltage, temperature, etc. The fusion of sensory data from heterogeneous platforms, i.e., multimodal fusion, is for the purpose that complementary information could be extracted from each modality, thus yielding a richer representation that could be used to achieve higher-quality intelligent FDP, compared to using only a single modality [165]. The efficient fusion of multimodal sensory data remains challenging for the community.

Early stage of multimodal fusion mainly are at data-level, i.e., representing the fused data in a lower-dimensional subspace, in which principal component analysis is commonly used. It is then extended to feature-based fusion that features extracted from each model for each modality is fused, and decision-based fusion which makes a weighted fusion decision for the outputs of those models [166]. For example, [167] use a coupling AE to find a joint feature between vibration and acoustic signals for health-state classification, and [168] propose to extract the multiscale features of vibration and torque signals through a three-stage feature fusion method for the fault diagnosis of bearings. In [169], a multimodal decision-fusion model is built to achieve comprehensive fault diagnosis for rotor-bearing systems.

As can be observed in the related literatures of multimodal fusion for intelligent FDP, current modalities used mostly are derived from similar mechanisms, such as acceleration signals and acoustic signals formed by vibration, and voltage and current signals formed by electronics. They are generally with the same data representation and can easily be fused through data transformations. The modalities derived from different mechanisms are merely used, for example the fusion of vibration signals and 2-D images, temperature signals and current signals, or even text descriptions and images. Therefore, there is still room for the fusion of these modalities to boost the performance and applicability of intelligent FDP in complex industrial systems.

### 6.4. Intelligent FDP Acceleration for Edge Implementation

Industrial IoT and AI have been playing highly significant roles in modern industrial systems, more and more sensors are installed, generating massive amounts of sensory data. With the increase in data scale, the response delay of data transmission and calculation cannot be guaranteed, which brings great challenges to the computing center-based industrial systems. Moreover, modern, intelligent FDP algorithms based on deep learning are generally computationally intensive, i.e., with huge parameters and deep architectures.

To tackle this problem, an emerging computing paradigm, edge computing, has been widely recognized as a promising solution [170]. In the edge computing paradigm, model training is performed by the center, and models are deployed and runs on the edge nodes, such as gateway, smart devices, and the way of bringing data and computation closer to where data are produced can help to save the response time and bandwidth, as well as energy consumption [171].

However, edge ends are always constrained by resources, which means their power supply and computing capability are limited and heavy deep learning models can hardly adapt to these platforms. Therefore, it brings great challenges to the intelligent FDP algorithms in turn. Models that are computationally lightweight and of high accuracy are preferred for the edge implementation [172]. In the field of computer vision, the lightweight design of deep learning models has been a hot research spot for edge implementation, and typical methods are network pruning [173] and knowledge distillation [174]. Currently, some pioneer work [175,176] has been conducted and shown promising results for intelligent FDP on edge ends.

## 7. Conclusions

The diagnosis and prognosis of faults are important for the operation of industrial systems. This paper mainly reviews the development of deep learning techniques in intelligent FDP for industrial systems. The tasks of fault diagnosis and fault prognosis are firstly defined mathematically. An overview of deep learning architectures that are commonly used for intelligent FDP are then summarized. To be specific, the architectures of DBN, CNN, AE, RNN, GAN, Transformer, and GNN are introduced, along with their applications. Finally, we prospect four future directions from the aspects of data imbalance, compound fault type, multimodal data fusion, and edge implementation, and possible solutions are also provided. This survey is expected to comprehensively present the development of deep learning techniques used in intelligent FDP for industrial systems and provide possible guidelines for the research in the community.

Early detection, isolation, and identification of different faults enabled with DL techniques will help to greatly improve the efficiency, reliability, and repeatability of industrial systems. With the fast development and evolution of DL and related techniques, in near future many fundamental problems, such as the mentioned open challenges, are very likely to be addressed. As for the research trends, the borders between different DL architectures are being broken down and a hybrid architecture that takes both advantages is expected to produce better flexibility and performance. In addition, physics-informed DL techniques based on the physical characteristics and related physical models of the industrial system will be an important future direction.

## Figures and Tables

**Table 1:** Recent publications of intelligent FDP methods based on AEs.

Type	Reference	Year	Method	Object
	[39]	2019	A stacked AE for compressing the feature depth	high-voltage circuit breakers
Standard AE	[47]	2020	1-D residual convolutional AE for learning features from vibration signals directly in an unsupervised-learning way	machinery
	[40]	2022	AE with adaptive Morlet wavelet to establish accurate mapping hidden in the fused health index	aeroengine
	[38]	2022	Stacked AE to establish an accurate non-linear mapping between the raw data and different fault states	rotating machinery
Denoising AE	[41]	2018	Stacked denoising AE to extract useful feature and reduce the dimension of vibration signal to 2 or 3 dimensions	bearing
Sparse AE	[42]	2022	Sparse representation convolutional AE to extract impulsive components of vibration signals	rotating machinery
Sparse denoising AE	[48]	2019	A sparse stacked denoising AE is proposed for feature extraction	bearing
Variational AE	[43]	2022	A convolutional variational AE with attention mechanism providing better spatial distributions of features	aeroengine
Contractive AE	[44]	2018	Stacked contractive AE for automatic robust features extraction	rotating machinery

**Table 2:** Some of recent intelligent FDP methods based on GANs.

Type	Reference	Year	Method	Object
Data generation	[56]	2019	GAN is used to refine the rough fault data more similar with real data.	wind turbine
	[57]	2019	An auxiliary classifier GAN-based framework to learn from mechanical sensor signals and generate realistic one-dimensional raw data.	induction motor
	[58]	2020	GAN to generate new samples similar to the simulation and measurement fault samples in order to enlarge datasets.	bearing
	[59]	2021	GANs is used to acquire abundant synthetic samples generated from the simulation and measurement samples, which aims to expand fault samples.	rotor-bearing systems
	[60]	2021	DCGAN is employed to produce new face-portraits of the nominal and failure behaviors.	ball-bearing joints
	[53]	2022	GAN to enhance the deep features of real signals.	rolling bearings
	[61]	2022	GAN uses available time series degradation data to generate synthetic degradation data.	bearing
	[62]	2022	A Wasserstein conditional GAN constrain the data generation characteristics to improve the validity of data.	rolling bearings
Local domain FD	[63]	2020	A semi-supervised multi-scale convolutional GAN to learn discriminativity from unlabeled data.	rolling bearings
	[64]	2022	Stepwise GAN trains multistage with unlabeled normal data and fuses multi-source information at feature level and aggregating neighboring information at decision level	liquid rocket engine
Cross domain FD	[58]	2020	Domain adversarial transfer network exploits task-specific feature learning networks and domain adversarial training techniques for handling large distribution discrepancy across domains.	rotating machinery
	[55]	2021	A deep transfer learning model based on an adversarial learning strategy to effectively separate multiple unlabeled new fault types.	mechanical equipment
	[65]	2022	A one-class GAN based on semi-supervised learning to learn one-class latent knowledge for dealing with multiple semi-supervised fault diagnosis tasks.	industrial robot

**Table 3:** A compilation of recent intelligent FDP methods based on DBNs.

Purpose	Reference	Year	Method	Object
Classification	[74]	2019	Convolutional DBN based on Fisher parameter optimization	rolling bearings
	[79]	2020	DBN optimized by quantum-inspired differential evolution	rolling bearings
	[80]	2022	DBN classifies features from wavelet energy entropy	robot joint bearing
	[81]	2022	Gaussian convolutional DBN for classification	rotor bearing system
Feature Extraction	[82]	2020	Multi-scale cascading DBN for feature extraction	rotating machinery
	[68]	2020	Convolutional DBN for feature extraction	reciprocating compressors
	[83]	2022	Dilated convolution DBN to extract transferable characteristics	roller bearing
Feature Fusion	[71]	2019	DBN for feature fusion and classification	wind turbine gearbox
	[84]	2022	DBN fuses multivariables for parameter estimation	deep-sea human occupied vehicle
Index Regression	[66]	2019	DBN to construct health indicator for RUL prediction	aircraft engine
	[85]	2020	Median filtering DBN to extract health indicator	bearings
	[86]	2021	DBN to eliminate health indicator curve oscillation	bearings
Pretraining	[72]	2015	DBN to pretrain multilayer neural network	gearbox

**Table 4:** Recent publications of intelligent FDP methods based on RNNs.

Type	Reference	Year	Purpose	Method	Object
basic RNN	[93]	2020	Fault prediction	A fully connected RNN to predict faults from signal data dimensionally reduced.	nuclear power machinery
	[94]	2022	Fault diagnosis	RNN to identify different relevant types of faults, based on the past 24h of satellite measurements without on-site sensors.	photovoltaic systems
GRU	[95]	2020	RUL estimation	GRU to construct health indicator from sensitive fetures.	rolling element bearings
	[96]	2021	Fault diagnosis	GRU to exploit temporal information of time-series data and learn representative features from constructed signal images.	rotating machinery
	[87]	2021	Fault diagnosis	RNN with GRU and LSTM to capture the hidden patterns of vibration time series.	power transformer
	[88]	2022	Fault diagnosis	GRUs to understand whether data in a time series is crucial enough to preserve or forget.	bearings of wind turbines
LSTM	[97]	2019	Fault diagnosis	LSTM to capture long-term dependencies through recurrent behaviour.	wind turbines
	[98]	2020	RUL estimation	A LSTM model fuses multi-sensor monitoring signals to discover the hidden long-term dependencies among sensor time series signals.	turbofan engine
	[34]	2020	Fault diagnosis	LSTM learns long-term dependencies from the concatenated feature and operation state indicator of the equipment.	aircraft turbofan engines
	[91]	2021	RUL estimation	Convolution-based LSTM to capture long-term dependencies and extract features from the time-frequency domain at the same time.	rotating machinery
	[90]	2021	RUL estimation	Dual LSTM to characterize both long and short-term dependenciesfrom historical information.	turbofan engine
	[99]	2022	Fault diagnosis	CNN to determine spatial correlations between two measurements within one time step, and LSTM to identify temporal dependencies between two adjacent time steps.	planetary gearbox

**Table 5:** A list of recent intelligent FDP methods based on CNNs.

Type	Reference	Year	Method	Object
Camera sensors	[100]	2019	CNN for feature extraction and classification	cooling radiator
	[101]	2020	CNN extracts fault features from infrared thermal images	rotating machinery
	[102]	2021	Mask rcnn for detection	power transformers
Signals to images	[103]	2019	Wavelet transform is adopted to extract 2-D time-frequency features from raw 1-D vibration signals	gearboxes
	[104]	2020	Continuous wavelet transform (CWT) converts signals into images	aeroengine control system
	[105]	2020	Sensor signals are converted to time-frequency distribution by wavelet transform	induction motor
	[106]	2021	1-D vibration signals are converted to 2-D grayscale vibration images	rolling element bearing
	[107]	2021	Vibration signals are first transformed into angular domain and then converted to corresponding envelope and squared envelope spectrum features, which are fused into RGB color image form	mechanical rotating components
	[108]	2022	CWT converts the vibratory time-series signals to the scalogram feature images	induction motors
	[109]	2022	A conversion method based on principal component analysis is applied to fuse multisignal data into three-channel RGB images	mechanical manufacturing systems
1-D CNN	[110]	2018	1-D CNN learns features adaptively from raw mechanical data without prior knowledge	motor bearing
	[111]	2019	Adaptive 1-D CNN for real-time and highly accurate circuit monitoring system	modular multilevel converter
	[112]	2020	Multi-attention 1-D CNN to diagnose faults	rolling bearing
	[113]	2021	1-D CNN to learn feature from the high-frequency components	high-speed train bogie
	[114]	2022	1-D CNN to establish model for fault diagnosis	UAV rotor
	[115]	2022	Multi-level features fusion 1-D CNN for good performance of feature extraction on vibration signals	bearing

**Table 6:** Some of recent intelligent FDP methods based on a transformer.

Type	Reference	Year	Method	Object
Fault diagnosis	[130]	2021	Linear embedding sequence of signal patches is used as an input to a Transformer encoder, CNN is used as decoder and classifier.	bearing and gearbox datasets
	[128]	2022	A time-frequency Transformer model with a new tokenizer and encoder module to extract effective abstractions from the time-frequency representation of vibration signals.	bearing
	[131]	2022	The weight parameters of self-extracted features of SPBO-SDAE network are optimized through the self-attention mechanism of transformer to retain the target features and filter the redundant features.	rotating machinery
	[132]	2022	A lightweight transformer based on convolutional embedding and linear self-attention to deal with the challenges of limited samples, noise interference, and lightweight.	rotating machinery
	[133]	2022	Convformer-NSE to extract robust features that integrate both global and local information under heavy noise.	gearbox systems
	[127]	2022	Time series transformer with a tokens sequences generation method handling data in 1D format.	rotating machinery
	[134]	2022	Transformer is built to extract temporal features.	electromagnetic systems
	[135]	2022	Transformer architecture is employed to diagnose the simultaneous faults with time-series data.	on-site air handling unit
Fault prediction	[136]	2021	As a variant of transformer, Informer is used for Long sequence time-series prediction.	nuclear power valves
	[137]	2022	Informer is introduced to solve the problem of error accumulation caused by the conventional methods of time series forecasting of motor bearing vibration.	bearing
RUL prediction	[138]	2022	A self-attention module is designed by adopting the attention mechanism into ConvLSTM cell to focus on the degraded data that is beneficial to the prediction result, and suppressing less useful ones.	bearing
	[139]	2022	Convolutional transformer combines the global context capturing of attention mechanism with the local dependencies modeling of convolutional operation	bearing

**Table 7:** Some of recent intelligent FDP methods based on GNNs.

Type	Reference	Year	Method	Object
GCN	[148]	2020	A deep GCN based on graph theory transforms data into graphs of geometric structures with weights representing the similarity between connected vertices.	roller bearings
	[149]	2021	Semi-supervised GCN constructs all samples into an undirected and weighted k-nearest neighbor graph, which is trained using both labeled and unlabeled samples.	rotating machinery
	[150]	2021	GCN incorporates the weighted horizontal visibility graph to transform time series to graph data, and uses graph isomorphism network to learn the graph representation and perform fault classification.	bearing
	[151]	2021	GCN decomposes signals to present frequency feature as graph and extract the features of points with a large span of the defined graph samples.	wind turbine
	[144]	2021	A structure analysis-based GCN integrates the measurement and the prior knowledge of the system of interest and introduces a weight coefficient to adjust their influence.	rectifier
	[152]	2022	Multi-scale cluster-GCN is proposed to learn the representation feature extracted by AE layer.	gearbox and bearing
	[153]	2022	Edge connections of the input static graph are updated according to the relationship among high-level features extracted by GCN.	rotating machinery
GAT	[145]	2021	A semi-supervised conditional random field-based GAT learns the effective node representations and models the label dependency through assigning adaptive weights to different neighbors.	motor
	[154]	2022	A triplet metric driven multi-head GNN combines deep metric learning and improves triplet loss to convert signals into graph structure, and introduces multi-head attention to reduce interference of heterogeneous vertices.	rolling bearing
GAE	[146]	2022	Graph dynamic AE uses graph convolution to avoid the dimensionality increase problem of classic dynamic methods, and a weighted adjacency matrix to adaptively assign weights to the temporal samples.	Tennessee Eastman process
	[155]	2022	Sparse AE and GNN are combined to effectively capture inter-dependencies in high-dimensional sensor data with few anomalies.	cyber-physical systems

## Data Availability

Not applicable.
